# Cleft Candidate Genes and Their Products in Human Unilateral Cleft Lip Tissue

**DOI:** 10.3390/diseases9020026

**Published:** 2021-04-07

**Authors:** Mārtiņš Vaivads, Ilze Akota, Māra Pilmane

**Affiliations:** 1 Institute of Anatomy and Anthropology, Riga Stradins University, Kronvalda Boulevard 9, LV-1010 Riga, Latvia; Mara.Pilmane@rsu.lv; 2 Department of Oral and Maxillofacial Surgery, Riga Stradins University, 16 Dzirciema Street, LV-1007 Riga, Latvia; Ilze.Akota@rsu.lv; 3 Cleft Lip and Palate Centre, Institute of Stomatology, Riga Stradins University, 20 Dzirciema Street, LV-1007 Riga, Latvia

**Keywords:** cleft lip, cleft candidate genes, gene proteins

## Abstract

Cleft lip and palate are common congenital pathologies that affect the human population worldwide. The formation of cleft lip is associated with multiple genes and their coded proteins, which regulate the development of craniofacial region, but the exact role of these factors is not always clear. The use of morphological studies for evaluation of human cleft-affected tissue has been limited because of insufficiency of available pathological material. The aim of this study was to detect and compare the immunohistochemical expression of cleft candidate gene coded proteins (DLX4, MSX2, HOXB3, SHH, PAX7, SOX3, WNT3A, and FOXE1) in the non-syndromic unilateral cleft lip patient tissue and control group tissue. A semiquantitative counting method was used to evaluate the tissue in biotin-streptavidin-stained slides. Statistically significant differences between the patient and control groups were found for the number of immunoreactive structures for SHH (*p* = 0.019) and FOXE1 (*p* = 0.011) in the connective tissue and SOX3 (*p* = 0.012) in the epithelium. Multiple statistically significant very strong and strong correlations were found between the immunoreactives in cleft-affected tissue. These significant differences and various correlations indicate that multiple morphopathogenetic pathways are possibly involved in unilateral cleft lip pathogenesis. Therefore, we further discuss these possible interactions.

## 1. Introduction

Cleft lip and palate are one of the most common congenital pathologies in the human population. Data may differ from one geographical location and population to another but the global incidence of cleft lip and palate is approximately 1 in 500–2500 newborns. Data from the United States shows that more than 60% of craniofacial clefts affect the lip region and isolated cleft lip takes up to 10–30% of all orofacial clefts with unilateral cleft lip being more common than the bilateral cleft lip [1].

Non-syndromic cleft lip and palate pathogenesis involves the dysregulated expression of multiple genes. These genes play an important role in regulating the development of the craniofacial region. Dysregulation of these genes is associated with the development of craniofacial clefts but the exact mechanisms are not always clearly known. These genes and their coded proteins have been studied by mostly using animal models but studies on human cleft tissues are limited because of ethical concerns and the lack of available material [2].

The distal-less homeobox (DLX) genes belong to homeodomain-containing transcription factors that are necessary during the embryonic development in processes of neurogenesis and limb pattern formation. DLX genes are expressed in the primordia of the developing facial region in different patterns both regionally and temporally [3]. DLX4 has been associated with formation of orofacial clefts in humans where a DLX4 polymorphism (c.546_546delG, predicting p.Gln183Argfs*57) was identified in a mother and her child with a bilateral cleft lip and palate [4]. Recently multiple DLX4 gene polymorphisms have been analyzed within the Han Chinese population but no significant correlation with the formation of nonsyndromic orofacial clefts were clearly identified [5].

Muscle segment homeobox gene 2 (MSX2) is a member of the family of divergent homeobox-containing genes. MSX2 gene mutations have been associated with formation of different cleft lip and palate phenotypes [6]. MSX2 together with MSX1 is detectable in the developing craniofacial skeleton, including the maxilla, mandible, teeth germs and Meckel’s cartilage [7]. Msx2-null mutant mice show a phenotype characterized by defective amelogenesis, tooth root dysmorphology and other abnormalities in teeth [8], skull ossification defects, persistent calvarial foramen and defects in endochondral ossification [9]. In humans MSX2 gene mutations are associated with Boston type craniosynostosis [10,11].

Homeobox B3 (HOXB3) gene encodes a transcription factor that regulates the migration process of neural crest stem cells. It also plays a role in the formation of pharyngeal apparatus and derivates of the 3rd and 4th pharyngeal arch pouches, including the parathyroid glands and thymus [12]. HOXB3 together with HOXA3 and HOXD3 regulate the migration of the thymus and parathyroid glands to their correct positions. Disruption in the function of these genes in mice showed defective formation of these throat organs [13].

The sonic hedgehog (SHH) gene encodes a protein that is an essential part of Shh signaling pathway, which is necessary for proper craniofacial development, especially the formation of the palate and frontonasal development. Disruption of the SHH pathway is associated with orofacial clefts [14]. SHH signaling promotes cranial neural crest cell proliferation during the formation of the upper lip and disruption in this pathway can lead to cleft lip development [15]. SHH also is important in formation of the cleft palate. SHH is expressed in epithelial rugae and maintains epithelial–mesenchymal interactions necessary for the formation of the secondary palate. Excessive Shh signaling causes downregulation of Wnt/bone morphogenetic protein (BMP) signaling pathway, inducing the formation of cleft palate [16].

Wingless-Type MMTV Integration Site Family, Member 3A (WNT3A) together with other WNT genes have been associated with formation of the cleft lip and palate [17]. WNT3A has been detected in different craniofacial locations—in the primary palate, secondary palate and developing upper lip and regulate regional specification of the developing face [18]. In mouse models transient Wnt signaling activity has been detected in subectodermal dermal progenitors and cranial bone progenitors, suggesting an instructive role in formation of these craniofacial structures. Persistent Wnt signaling activity can be detected during the development of teeth [19].

Forkhead box protein E1 (FOXE1) is a transcription factor, which contains a DNA-binding forkhead domain. FOXE1 dysfunction is associated with the formation of the cleft palate and dysgenesis of the thyroid gland in mouse models, but in humans homozygous FOXE1 mutations cause Bamforth–Lazarus syndrome characterized by cleft palate and congenital hypothyroidism [20]. FOXE1 expression has been detected in the epithelium, which will undergo fusion between the medial nasal and maxillary processes. Cleft lip with or without cleft palate and isolated cleft palate phenotypes have been associated with FOXE1 mutations [21].

Paired box 7 (PAX7) is a transcription factor, which is associated with formation of cleft lip and palate [22]. It is expressed in the developing craniofacial region within the neural crest cells [23]. PAX7 gene polymorphisms have been significantly associated with cleft lip and palate in a genome wide population association studies [24,25]. PAX7 together with PAX3 gene regulates the development of craniofacial region and mice with impaired PAX3/PAX7 genes exhibit growth arrest of cranial neural crest cells in the frontonasal region, leading to formation of frontal cleft face in mice models [26].

SRY-Box Transcription Factor 3 (SOX3) gene expression has been detected in neural crest cells. SOX3 has been found to be involved in the earliest formation of the pharyngeal structures, including the formation of pharyngeal pouches, which segments the pharyngeal region and individualizes each pharyngeal arch [27]. Multiple different SOX genes are expressed within the developing palatal tissues and developing tooth primordia in mice but SOX3 expression in these areas is limited or it is not detectable [28,29].

In this study these following cleft candidate gene coded proteins were analyzed in the cleft patient and control groups—DLX4, MSX2, HOXB3, SHH, WNT3A, FOXE1, PAX7 and SOX3. These genes and their coded proteins were selected for this study, because of their involvement in the formation of the lip and the craniofacial region, development of surface epithelium and underlying ectomesenchymal tissue. The aim of this research was to study the appearance and distribution and of these proteins within the patient and control group tissue and detecting intercorrelations between these factors within the cleft affected tissue.

## 2. Materials and Methods

The study group included 10 patients with non-syndromic unilateral cleft lip (soft cleft tissues were taken during cleft surgery) and 5 controls without a cleft lip or palate with tissue samples taken from frenula labii superioris during labial frenectomy due to the correction of low attached upper lip frenulum. The control group was selected based on the availability of tissue material from the upper lip region mucosa, which was not affected by orofacial clefts, inflammation or any other pathology. All tissue samples from both groups were taken with voluntarily agreement from patients to donate the tissue samples for research. The samples were taken in Cleft Lip and Palate Centre of the Institute of Stomatology of Riga Stradins University. The study was conducted in the Department of Morphology of Riga Stradins University. The study protocol was approved by the ethics committee of Riga Stradins University in May 2003 and September 2020 (22.05.2003; 24.09.2020; Nr.6-1/10/11).

Standard biotin and streptavidin immunohistochemical method was used for the detection of the mentioned gene coded proteins [30]. The fixation of tissue samples was performed in 2% formaldehyde and 0.2% picric acid in 0.1 M phosphate-buffer (pH 7.2). Afterwards washing was done in phosphate-buffered saline containing 10% saccharose for 12 h, embedded in paraffin and cut into 6–7 μm thick sections. Afterwards deparaffinization was performed and slide staining was done with the biotin-streptavidin immunohistochemical method to detect the presence of proteins with antibodies for DLX4 (orb160775, 1:100, Biorbyt Ltd., Cambridge, UK), MSX2 (ab22606, 1:100, Abcam, Cambridge, UK), HOXB3 (sc28606, 1:100, Santa Cruz Biotechnology, Dallas, TX, USA), SHH (LS-C49806, 1:100, LifeSpan BioSciences, Inc., Seattle, WA, USA), WNT3A (ab19925, 1:800, Abcam, Cambridge, UK), FOXE1 (ab5080, 1:500, Abcam, Cambridge, UK), PAX7 (ab55494, 1:100, Abcam, Cambridge, UK) and SOX3 (orb158460, 1:100, Biorbyt Ltd., Cambridge, UK).

Slide visual illustration was performed with Leica DC 300F digital camera and image processing and analysis was done with Image Pro Plus software (Media Cybernetics, Inc., Rockville, MD, USA).

To record and evaluate the relative frequency of indices detected by using the immunohistochemical method, a semiquantitative counting method was used for non-parametric evaluation of slides (Table 1) [31]. The factor appearance frequency of positively stained cells was analyzed in five visual fields of each section with slide analysis by light microscopy.

Data analysis was performed using descriptive and analytical statistical methods. The count of factor positive cells per each of the 5 visual fields, median value and standard deviation calculation was performed. Further Spearman’s rank correlation analysis was done and statistical significance was determined with a Mann–Whitney U test between the controls and patient group. The data statistical analysis was done with the statistical program SPSS Statistics (version 26.0, IBM Company, Chicago, IL, USA). The semiquantitative count of immunoreactive cells is shown as median values. Two-tailed *p* values < 0.05 were considered statistically significant for all statistical calculations.

## 3. Results

The presence of gene coded proteins DLX4, MSX2, HOXB3, SHH, PAX7, SOX3, WNT3A and FOXE1 in patient and control group tissue material was rather variable.

**Figure 1:**
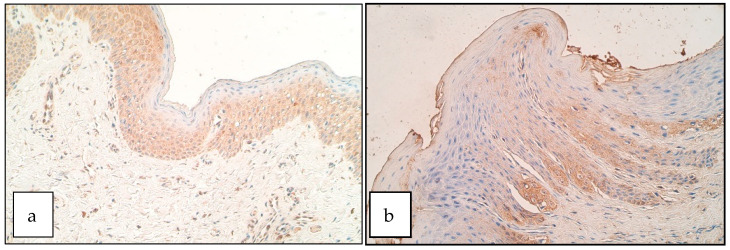
Immunoreactive structures for DLX4. (**a**) Moderate DLX4 positive structures in the patient epithelium, DLX4 IHM, 200×. (**b**) Moderate DLX4 positive structures in the control group, DLX4 IHM, 200×.

In the control group the number of DLX4 positive structures ranged from no positive structures (0) to moderate to numerous (++/+++) in the epithelium and a few positive structures (+) to moderate to numerous (++/+++) in the connective tissue (Figure 1b). The number of DLX4 positive structures in the epithelium of patients ranged from no positive structures (0) to numerous (+++) positive structures in the visual field. In the connective tissue the number of DLX4 positive cells (mainly macrophages and some fibroblasts) ranged from a rare occurrence (0/+) to moderate to numerous (++/+++) (Figure 1a). There was no statistically significant difference between the patient and control group for the number of DLX4 positive structures in the epithelium (U = 20.0, *p* = 0.594) and connective tissue (U = 17.5, *p* = 0.371).

**Figure 2:**
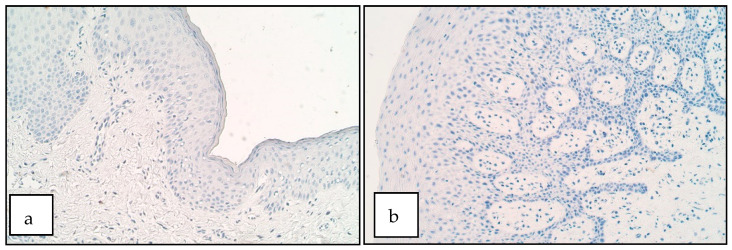
Immunoreactive structures for MSX2. (**a**) No positive structures for MSX2 in patient epithelium and connective tissue. MSX2 IHM, 200×. (**b**) No positive structures for MSX2 in control epithelium and connective tissue, MSX2 IHM, 200×.

MSX2 positive cells were not found (0) in the epithelium of both controls and patients. In the connective tissue of both patients and controls the number of MSX2 positive structures ranged from no positive structures (0) to barely detectable (0/0/+). MSX2 was barely detectable only in couple of patients in a small number of macrophages (Figure 2a,b). There was no statistically significant difference between the patient and the control group for the number of MSX2 positive structures in the epithelium (U = 25.0, *p* = 1.000) and connective tissue (U = 22.5, *p* = 0.768).

**Figure 3:**
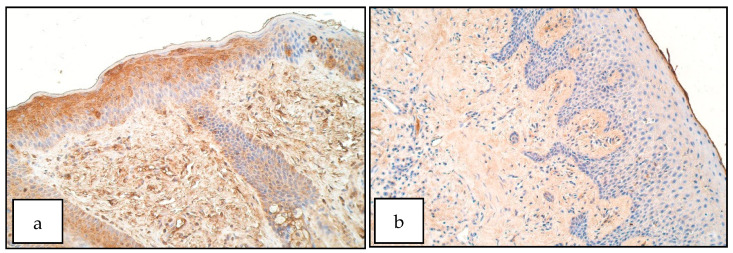
Immunoreactive structures for HOXB3. (**a**) Moderate to numerous HOXB3 positive structures both in patient epithelium and connective tissue, HOXB3 IMH, 200×. (**b**) A few HOXB3 positive structures in control epithelium, HOXB3 IMH, 200×.

In the control group the number of HOXB3 positive structures ranged from a few (+) to moderate (++) in the epithelium and few to moderate (+/++) to moderate (++) in the connective tissue (Figure 3b). For HOXB3 the number of positive structures in the epithelium of the patient group ranged from no positive structures (0) to numerous (+++) and in the patient connective tissue from a few (+) to numerous to abundant (+++/++++). HOXB3 in patient connective tissue was detectable in macrophages, fibroblasts and endothelial cells (Figure 3a). There was no statistically significant difference between the patient and control group for the number of HOXB3 positive structures in the epithelium (U = 11.0, *p* = 0.099) and connective tissue (U = 21.0, *p* = 0.679).

**Figure 4:**
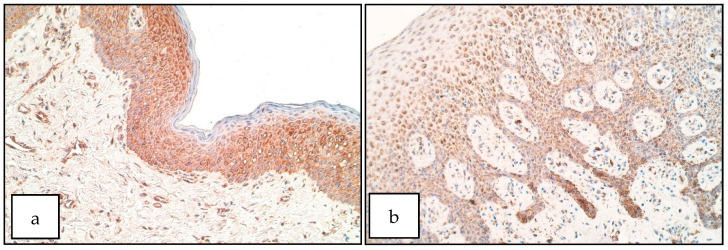
Immunoreactive structures for SHH. (**a**) Moderate to numerous SHH positive structures in patient epithelium, SHH IMH, 200×. (**b**) Moderate SHH positive structures in control epithelium, SHH IMH, 200×.

In the control group the number of SHH positive structures ranged from a few (+) to few to moderate (+/++) in the epithelium and moderate (++) to moderate to numerous (++/+++) in the connective tissue (Figure 4b). The number of SHH positive cells in patient group ranged from no positive structures (0) to numerous to abundant (+++/++++) in the epithelium (Figure 4a) and from moderate (++) to numerous (+++) SHH containing macrophages and fibroblasts in the connective tissue. There was no statistically significant difference between the patient and control group for the number of SHH positive structures in the epithelium (U = 10.0, *p* = 0.075) but a significant increase of SHH positive structures was observable in patient connective tissue compared to controls (U = 6.5, *p* = 0.019).

**Figure 5:**
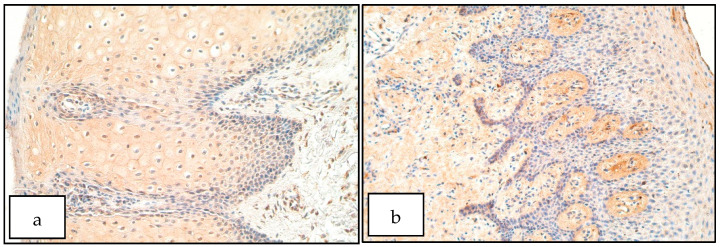
Immunoreactive structures for PAX7. (**a**) Few to moderate PAX7 positive structures in the patient epithelium, moderate PAX7 positive structures in the patient connective tissue, PAX7 IHM, 200×. (**b**) Few to moderate PAX7 positive structures in control group epithelium, moderate positive structures in control connective tissue, PAX7 IHM, 200×.

In the control group the number of PAX7 positive structures ranged from a few (+) to few to moderate (+/++) in the epithelium and a few (+) to moderate to numerous (++/+++) in the connective tissue (Figure 5b). For PAX7 in the patient group the number of positive structures ranged from no positive structures (0) to moderate to numerous (++/+++) in the epithelium (Figure 5a) and from rare occurrence (0/+) to moderate to numerous (++/+++) in the connective tissue, especially in macrophages. There was no statistically significant difference between the patient and the control group for the number of PAX7 positive structures in the epithelium (U = 10.0, *p* = 0.075) and connective tissue (U = 14.5, *p* = 0.206).

**Figure 6:**
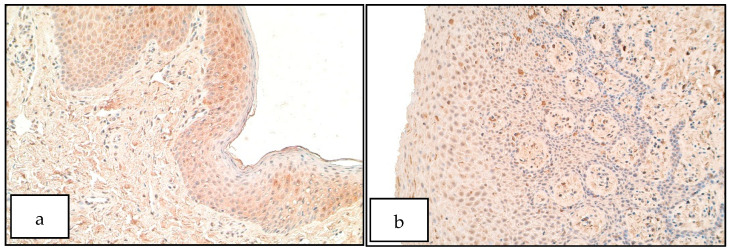
Immunoreactive structures for SOX3. (**a**) Moderate number of SOX3 positive structures in the patient epithelium and connective tissue. SOX3 IHM, 200×. (**b**) Few to moderate positive structures in the control group epithelium, SOX3 IHM, 200×.

In the control group the number of SOX3 positive structures ranged from a few (+) to moderate (++) in the epithelium and from a few (+) to moderate (++) in the connective tissue (Figure 6b). The number of SOX3 positive structures in the patient epithelium ranged from no positive structures (0) to numerous (+++) (Figure 6a). In the patient connective tissue the number SOX3 positive structures ranged from few to moderate (+/++) to moderate to numerous (++/+++) positive structures. There was a statistically significant increase of SOX3 positive cells in patient epithelium compared to controls (U = 7.0, *p* = 0.028), but no statistically significant difference was found between patient and control connective tissue (U = 15.0, *p* = 0.254).

**Figure 7:**
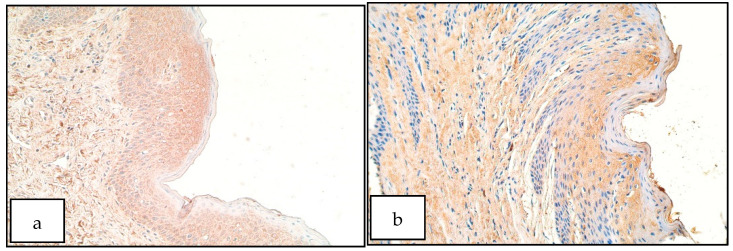
Immunoreactive structures for WNT3A. (**a**) Moderate to numerous WNT3A positive structures in the patient epithelium and moderate number of WNT3A positive structures in the connective tissue, WNT3A IMH, 200×. (**b**) Moderate number of WNT3A positive structures in the control epithelium, WNT3A IMH, 200×.

In the control group the number of WNT3A positive structures ranged from a rare occurrence (0/+) to moderate (++) in the epithelium and a few (+) to moderate (++) in the connective tissue (Figure 7b). The number of WNT3A positive structures in the patient epithelium ranged from a rare occurrence (0/+) to numerous (+++) and from a rare occurrence (0/+) to moderate (++) in the connective tissue (Figure 7a). There was no statistically significant difference between the patient and the control group for the number of WNT3A positive structures in the epithelium (U = 21.0, *p* = 0.679) and connective tissue (U = 23.0, *p* = 0.859).

**Figure 8:**
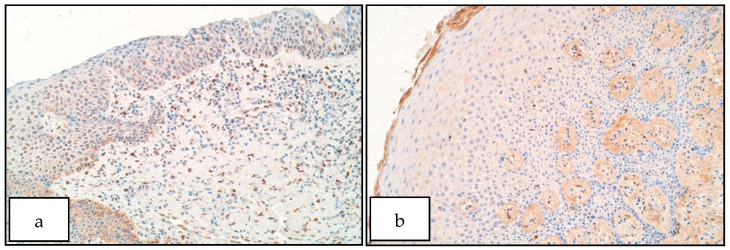
Immunoreactive structures for FOXE1. (**a**) Moderate number of FOXE1 positive structures in patient epithelium and connective tissue, FOXE1 IMH, 200×. (**b**) A few FOXE1 positive structures in control epithelium, FOXE1 IMH, 200×.

In the control group the number of FOXE1 positive structures ranged from a rare occurrence (0/+) to moderate (++) number in the epithelium and from a rare occurrence (0/+) to few to moderate (+/++) in the connective tissue (Figure 8b). For FOXE1 in the patient group the number of positive structures ranged from no positive structures (0) to moderate to numerous (++/+++) in the epithelium, especially in the basal cell layer (Figure 8a). In the connective tissue the number of FOXE1 immunoreactive structures ranged from no positive structures (0) to moderate to numerous (++/+++) positive structures, more prominently in macrophages. There was no statistically significant difference between the patient and control group for the number of FOXE1 positive structures in the epithelium (U = 14.0, *p* = 0.206) but a significant increase of SHH positive structures was observable in patient connective tissue compared to controls (U = 5.0, *p* = 0.013).

The median values for the semiquantitative evaluation of the number of immunoreactive structures for DLX4, MSX2, HOXB3, SHH, PAX7, SOX3, WNT3A and FOXE1 and subsequent Mann–Whitney U test values and *p*-values are summarized in (Table 2 and Table 3). More detailed information about the semiquantitative evaluation of the number of immunoreactive structures in 5 visual fields is available in the Supplementary Material.

In the statistical analysis the Mann–Whitney U test showed a statistically significant (*p* < 0.05) increase of number of SHH immunoreactive cells in patient connective tissue compared to controls (*p* = 0.019), a significant increase of number of FOXE1 immunoreactive cells in patient connective tissue compared to controls (*p* = 0.011), a statistically significant increase of number of SOX3 immunoreactive cells in the patient epithelium compared to controls (*p* = 0.023). No other statistically significant differences between patient and control groups were detected.

Spearman correlation coefficient calculation showed statistically significant correlations for the following factors in the patient group. In the patient epithelium very strong correlation was seen between the number of SHH positive cells in the patient epithelium and the number of FOXE1 positive cells in the patient epithelium (Spearman’s rho = 0.918, *p* < 0.001). Strong correlation was seen between the number of HOXB3 positive cells in the patient connective tissue and the number of SOX3 positive cells in the patient epithelium (Spearman’s rho = 0.817, *p* = 0.004), between the number of HOXB3 positive cells in the patient epithelium and the number of PAX7 positive cells in the patient epithelium (Spearman’s rho = 0.677, *p* = 0.032) and between the number of DLX4 positive cells in the patient epithelium and the number of WNTA3A positive cells in the patient epithelium (Spearman’s rho = 0.660, *p* = 0.038) (Table 4).

Significant Spearman’s rank correlation coefficient correlations were found between factors in patient epithelium and connective tissue. Very strong correlation was seen between the number of WNT3A positive cells in the patient epithelium and the number of WNT3A positive cells in the patient connective tissue (Spearman’s rho = 0.837, *p* = 0.003), and between the number of HOXB3 positive cells in the patient connective tissue and the number of SOX3 positive cells in the patient epithelium (Spearman’s rho = 0.817, *p* = 0.004). Strong correlation was seen between the number of HOXB3 positive cells in the patient epithelium and the number of HOXB3 positive cells in the patient connective tissue (Spearman’s rho = 0.727, *p* = 0.017), between the number of DLX4 positive cells in the patient epithelium and the number of DLX positive cells in the patient connective tissue (Spearman’s rho = 0.691, *p* = 0.027), between the number of HOXB3 positive cells in the patient epithelium and the number of PAX7 positive cells in the patient epithelium (Spearman’s rho = 0.677, *p* = 0.032) (Table 5).

## 4. Discussion

The formation of the cleft lip is a complicated interaction between multiple genes and signal pathways within the developing craniofacial region. The available information about the formation of unilateral cleft lip within humans is limited and mostly based on studies with animal models. In our study statistically significant differences were found between the number of immunopositive structures in patient and control groups for SHH, SOX3 and FOXE1.

SHH signaling is important for the correct formation of the craniofacial region. In our study in most cases a moderate to numerous number of SHH positive structures were detected in patient connective tissue and also in the epithelium. After evaluation of cleft affected tissues, a statistically significant increase in the number of SHH positive structures was observed in patient connective tissue compared to the control group, which may indicate the possible role of SHH in formation of cleft lip. As it is depicted in literature, enhanced SHH signaling activity within cranial neural crest cells of the developing facial region has been associated with improper lip fusion and the formation of cleft lip [32]. SHH pathway disruption has been associated with the formation of multiple craniofacial abnormalities, including cleft lip [33]. SHH activity is necessary for survival of cranial neural crest cells within the developing facial region [34].

In our study in most cases a moderate to numerous number of SOX3 positive structures were detected in patient epithelium and a moderate number of SOX3 positive structures were seen in the patient connective tissue. The patient group had significantly more SOX3 positive cells in the epithelium than the control group. This may indicate a possible interaction of SOX3 with the formation of cleft affected tissue. Information about the role of SOX3 involvement in the development of cleft lip is limited, but together with other SOX genes it contributes to the correct formation of facial structures during embryonic development [35]. SOX3 belongs to SoxB1 protein family together with SOX2 and SOX1, which have some overlap in function, and mostly regulate the development and proliferation of neural and sensory tissues, but also affect the developing craniofacial region [36].

In our study a statistically significant strong positive correlation was found between SOX3 immunopositive structures in the patient epithelium and HOXB3 positive structures in the patient epithelium, and a statistically significant very strong positive correlation was found between SOX3 positive structures in patient epithelium and HOXB3 positive structures in patient connective tissue, which may indicate a possible interaction between these factors within cleftaffected tissue. HOXB3 has been described affecting the proliferation of different cell lineages with research mainly focused on HOXB3 role in development of different cancer types and formation of metastases [37,38]. This possible interaction between SOX3 and HOXB3 might be involved with the regulation of cell proliferation within the cleft affected tissue.

In our study in most cases a moderate number of FOXE1 positive structures were detected in the patient connective tissue. After evaluation of cleft affected tissues, a statistically significant increase of FOXE1 positive structures was observed in patient connective tissue compared to the control group. FOXE1 has been previously associated with the formation of orofacial clefts. In other studies, FOXE1 activity has been described in the epithelium, which will undergo the fusion between the medial nasal and maxillary processes [21]. It is thought that FOXE1 gene variants together with other genes involved in facial development interact with each other and jointly increase the susceptibility of craniofacial defect formation, including cleft lip and palate [39]. FOXE1 affects cell growth and migration and has been described previously as a thyroid-specific factor, but it has been determined in mice models that FOXE1 is also involved with the development of craniofacial malformations like cleft palate [40,41].

Interestingly, a statistically significant very strong positive correlation was seen in the patient group between the number of FOXE1 positive structures in patient epithelium and between the number of SHH positive structures in the patient epithelium. This could be explained by the fact, that both SHH and FOXE1 function within interconnected signaling pathways during facial development. The indirect interaction between SHH and FOXE1 has been previously described in literature within the MSX1-BMP2-BMP4-SHH signaling loop during palatogenesis, where FOXE1 regulates MSX1 function, indirectly affecting SHH [42,43].

In the patient group macrophages contributed significantly to the number of SHH and FOXE1 positive structures. Enhanced Shh signaling in macrophages has been demonstrated in different studies analyzing tumor-associated macrophages, which are involved with the regulation of cell proliferation and local immune response within tumor tissue [44]. Our study results could indicate that SHH signaling may also affect the macrophage function and locally affect the tissue formation and proliferation in cleft-affected tissue. The information about the presence and the regulatory role of FOXE1 in macrophages is limited. FOXE1 could be involved with the regulation of SHH function. Interaction between FOXE1 and SHH has been described previously with the MSX1-BMP2-BMP4-SHH signaling loop [41]. One study analyzing the FOXE1 expression in papillary thyroid carcinoma showed increased number of macrophages in the periphery of tumor tissue, which may also indicate that FOXE1 might affect macrophage activity in tissue remodeling processes [45].

Statistically significant differences were not found between the number of immunopositive structures in the patient and control groups for DLX4, MSX2, HOXB3, PAX7 and WNT3A, although these factors still might be involved in the formation of a cleft lip.

At the end of the discussion, it can be emphasized that the formation of unilateral cleft lip is a complicated process with the involvement and interaction between multiple different factors. In our study our findings were characterized by the statistically significant increase of SHH, SOX3, and FOXE1 positive structures within the cleft affected tissue when compared to controls.

## 5. Conclusions

1. Increase of SHH positive structures in the unilateral cleft lip affected connective tissue could indicate a disruption of correct tissue formation in cleft-affected tissue, which could have affected proper lip fusion and the development of cleft lip.
2. Increase of SOX3 positive structures within patient epithelium could indicate a possible involvement of SOX3 in the formation of unilateral cleft lip possibly by affecting cell proliferation within the cleft-affected tissue.
3. Increase of FOXE1 positive structures within the cleft-affected connective tissue could indicate improper cell proliferation and formation within the developing lip region, resulting in remodeling processes within cleft-affected tissues.

## Figures and Tables

**Table 1:** Relative frequency designations of immunohistochemically determined gene proteins.

Designations Used	Explanations
0	No positive structures in the visual field
0/+	Rare occurrence of positive structures in the visual field
+	Few positive structures in the visual field
+/++	Few to moderate number of positive structures in the visual field
++	Moderate number of positive structures in the visual field
++/+++	Moderate to numerous positive structures in the visual field
+++	Numerous positive structures in the visual field
+++/++++	Numerous to abundant positive structures in the visual field
++++	Abundance of positive structures in the visual field

**Table 2:** Semiquantitative evaluation of immunoreactivity of DLX4, MSX2, HOXB3, SHH, PAX7, SOX3, WNT3A and FOXE1 in patient and control groups.

	DLX4		MSX2		HOXB3		SHH		PAX7		SOX3		WNT3A		FOXE1	
	E	CT	E	CT	E	CT	E	CT	E	CT	E	CT	E	CT	E	CT
Patients	++	++	0	0-0/+	++/+++	++	++/+++	++/+++	++	++	++/+++	++	+/++	+	++	++
Controls	++	+/++	0	0	+	++	+	+/++	+/++	+/++	+/++	+/++	+/++	+	+/++	+
U	20.0	17.5	25.0	22.5	11.0	21.0	10.0	6.5	10.0	14.5	7.0	15.0	21.0	23.0	14.0	5.0
*p*	0.594	0.371	1.000	0.768	0.099	0.679	0.075	0.019	0.075	0.206	0.028	0.254	0.679	0.859	0.206	0.013

Abbreviations: DLX4—distal-less homeobox 4; MSX4—muscle segment homeobox gene 2 protein, HOXB3—Homeobox B3, SHH—sonic hedgehog protein, PAX7—paired box 7, SOX3—SRY-Box Transcription Factor 3, WNT3A—Wingless-Type MMTV Integration Site Family, Member 3A, FOXE1—Forkhead box protein E1, E—epithelium; CT—connective tissue, Patients—median value in patient group, Controls—median value in control group, U—Mann–Whitney U test value, *p*—*p*-value, 0—no positive structures, 0/+—rare occurrence of positive structures, +—few positive structures, +/++—few to moderate number of positive structures, ++—moderate number of positive structures, ++/+++—moderate to numerous positive structures, +++—numerous positive structures, +++/++++—numerous to abundant structures, ++++—abundance of positive structures in the visual field.

**Table 3:** Median values of semiquantitative evaluation of DLX4, MSX2, HOXB3, SHH, PAX7, SOX3, WNT3A and FOXE1 in patient and control groups.

	DLX4		MSX2		HOXB3		SHH		PAX7		SOX3		WNT3A		FOXE1	
	E	CT	E	CT	E	CT	E	CT	E	CT	E	CT	E	CT	E	CT
P1	++	++	0	0/0/+	+++	+++	++	++	++	++/+++	+++	++	+/++	+	+/++	++
P2	0	0/+	0	0	0	+	0	++	0	0/+	0	++	0/+	0/+	0	0
P3	++	++/+++	0	0/0/+	+++	+++/++++	++	++/+++	++/+++	+++	+++	++	+++	++	+	++
P4	++	+/++	0	0	++/+++	++	++/+++	+++	++/+++	+++	++/+++	++	+	+	++	++
P5	++	++	0	0/0/+	++/+++	++/+++	+++	+/++	+++	++	++/+++	++/+++	0/+	0/+	++/+++	++
P6	++	+/++	0	0	++	+	+++/++++	++/+++	++	++	++/+++	++	+/++	++	++/+++	++
P7	++/+++	++/+++	0	0	+/++	+/++	+++	+++	++	++/+++	++	+/++	++	+/++	++/+++	++/+++
P8	++	++	0	0/0/+	++	++/+++	++/+++	+++	++	+/++	++/+++	++	++	+	++	++/+++
P9	+/++	++	0	0	+/++	++	++/+++	++/+++	++	++	++/+++	+/++	+	+	++/+++	+++
P10	+++	++/+++	0	0/0/+	++/+++	+/++	++/+++	++	++/+++	++	++	+/++	++/+++	++	++	++
Median (P)	++	++	0	0/0/+	++/+++	++	++/+++	++/+++	++	++	++/+++	++	+/++	+	++	++
C1	++	+	0	0	+	++	+	+/++	++	+/++	++	++	++	+	++	+
C2	++/+++	++/+++	0	0/0/+	++	+/++	++/+++	++	+/++	++/+++	++	+/++	+/++	+/++	+/++	+/++
C3	0	+/++	0	0	+	++	+	+/++	+	+	+	+	+/++	+	0/+	0/+
C4	+	+	0	0	+	++	++	++	+	+	+/++	++	+	+	++	+
C5	++	++	0	0/0/+	++	+/++	+	+/++	++	++	+/++	+/++	0/+	++	+	+
Median (C)	++	+/++	0	0	+	++	+	+/++	+/++	+/++	+/++	+/++	+/++	+	+/++	+

Abbreviations: DLX4—distal-less homeobox 4; MSX4—muscle segment homeobox gene 2 protein, HOXB3—Homeobox B3, SHH—sonic hedgehog protein, PAX7—paired box 7, SOX3—SRY-Box Transcription Factor 3, WNT3A—Wingless-Type MMTV Integration Site Family, Member 3A, FOXE1—Forkhead box protein E1, E—epithelium; CT—connective tissue, P1-P5—median value in each patient, C1-C5—median value in each control, Median (P)—median value in patient group, Median (C)—median value in control group, 0—no positive structures, 0/+—rare occurrence of positive structures, +—few positive structures, +/++—few to moderate number of positive structures, ++—moderate number of positive structures, ++/+++—moderate to numerous positive structures, +++—numerous positive structures, +++/++++—numerous to abundant structures, ++++—abundance of positive structures in the visual field.

**Table 4:** Spearman’s rank correlation coefficient correlations between different tissue factors in the patient group epithelium (r_s_—Spearman’s rho value).

Strength of Correlation	Correlations between Immunopositive Structures in Patient Group Epithelium	r_s_	*p*-Value
Very strong: 0.8–1.0	SHH and FOXE1	0.918	<0.001
Strong: 0.6–0.79	HOXB3 and SOX3	0.742	0.014
	HOXB3 and PAX7	0.677	0.032
	DLX4 and WNT3A	0.660	0.038

**Table 5:** Spearman’s rank correlation coefficient correlations between different tissue factors in the patient group epithelium and connective tissue (r_s_—Spearman’s rho value).

Strength of Correlation	Correlations between Immunopositive Structures in Patient Group Epithelium	r_s_	*p*-Value
Very strong: 0.8–1.0	WNT3A in epithelium and WNT3A in connective tissue	0.837	0.003
	SOX3 in epithelium and HOXB3 in connective tissue	0.817	0.004
Strong: 0.6–0.79	HOXB3 in epithelium and HOXB3 in connective tissue	0.727	0.017
	DLX4 in epithelium and DLX4 in connective tissue	0.691	0.027
	PAX7 in epithelium and HOXB3 in connective tissue	0.677	0.032

## Data Availability

The data used and/or analyzed during the current study is presented in the results section of this study.

## Supplementary Materials

The following are available online at https://www.mdpi.com/article/10.3390/diseases9020026/s1. Table S1: Semiquantitative evaluation of immunoreactivity of DLX4, MSX2, HOXB3, SHH, PAX7, SOX3, WNT3A, FOXE1 in patient and control groups in 5 visual fields; Table S2: Median values of semiquantitative evaluation of immunoreactivity of DLX4, MSX2, HOXB3, SHH, PAX7, SOX3, WNT3A, FOXE1 in patient and control groups.
